# Association Between Plant-Based Diets and Metabolic Syndrome in Zhejiang, China: A Cross-Sectional Study

**DOI:** 10.3390/nu17132159

**Published:** 2025-06-28

**Authors:** Mengyi Zhou, Ya Zhao, Mengjie He, Danting Su, Dan Han, Lichun Huang, Peiwei Xu, Ronghua Zhang

**Affiliations:** 1 School of Public Health, Hangzhou Medical College, Hangzhou 310059, China; 15058972559@163.com (M.Z.); 881012022123@hmc.edu.cn (Y.Z.); 2 Zhejiang Provincial Center for Disease Control and Prevention, Hangzhou 310051, China; mjhe@cdc.zj.cn (M.H.); dtsu@cdc.zj.cn (D.S.); dhan@cdc.zj.cn (D.H.); lchhuang@cdc.zj.cn (L.H.); pwxu@cdc.zj.cn (P.X.)

**Keywords:** plant-based diets, metabolic syndrome, cross-sectional study

## Abstract

**Background/Objectives:** Plant-based diets are associated with reduced chronic disease risk, though regional variations persist. In Zhejiang, China, where plant-based food intake is high, this study aimed to explore the association between plant-based diets and metabolic syndrome (MetS) in adults aged 35–75. **Methods:** This cross-sectional study utilized data from the 2024 Zhejiang Nutrition and Health Survey (ZJNHS). Three plant-based diet indices were calculated: the overall plant-based diet index (PDI), healthy plant-based diet index (hPDI), and unhealthy plant-based diet index (uPDI). Multivariate logistic regression models evaluated associations between diet index quintiles and MetS and its components. **Results:** Among the 4695 participants included in the study, 23.9% (*n* = 1122) had MetS. After adjusting for demographic and lifestyle factors, individuals in the highest uPDI quintile showed a significantly higher MetS risk compared to the lowest quintile (OR = 1.37, 95% CI: 1.08–1.73, *p*-trend = 0.013). Subgroup analyses revealed significant gender interaction (*p* for interaction < 0.001), with women exhibiting elevated MetS risk (OR = 1.03, 95% CI: 1.01–1.04, *p* < 0.001). For MetS components, the highest uPDI quintile was associated with increased risks of abdominal obesity (OR = 1.32, 95% CI: 1.05–1.66; *p*-trend = 0.032), elevated blood pressure (OR = 1.41, 95% CI: 1.12–1.78; *p*-trend = 0.003), and elevated fasting glucose (OR = 1.27, 95% CI: 1.01–1.59; *p*-trend = 0.037). **Conclusions:** Unhealthy plant-based foods are associated with increased MetS risk, particularly in women. Reducing intake of such foods, considering sex differences, and implementing precision nutrition interventions are essential.

## 1. Introduction

Metabolic syndrome (MetS) is a cluster of metabolic disorders characterized by abdominal obesity, hypertension, dysglycemia, elevated triglycerides, and reduced high-density lipoprotein cholesterol [1]. Recent global meta-analyses involving 28 million adults have demonstrated that the worldwide prevalence of MetS ranges from 12.5% to 31.4% [2], underscoring its status as a pressing global public health crisis with profound implications for population health. Concurrently, emerging evidence from national surveillance data highlights a concerning trend in China, where the burden of MetS has escalated significantly among adults—particularly females, who exhibit a 1.773-fold higher risk than males [3]. A subsequent cross-sectional study of Chinese women further highlighted that those aged 40–60 years face a significantly higher risk of metabolic abnormality clustering [4]. Beyond its established role in cardiovascular diseases (CVD) and type 2 diabetes (T2D) [5], emerging evidence links MetS to non-communicable diseases (NCDs) across endocrine, hepatic, and renal systems, amplifying the urgency for preventive interventions. In recent years, plant-based diets have garnered attention for their potential benefits in weight management, glycemic control, and cardiovascular risk factors [6,7,8]. Currently, the academic community has yet to reach a consensus on the definition of plant-based diets. Early research predominantly defined plant-based diets as patterns that prioritize plant-derived foods while substantially reducing or completely excluding animal-based food intake [9,10]. In contrast, contemporary studies emphasize that the essence of plant-based diets lies not only in minimizing animal products but also in discerning the quality of plant foods. To better assess the impact of plant-based diets on diseases, considering the quality differences among plant foods, Satija et al. developed the plant-based dietary index (PDI) [11,12]. This index allows for a more comprehensive evaluation by distinguishing between healthy and unhealthy plant-based foods, providing a more nuanced understanding of how plant-based eating patterns relate to health outcomes.

Unhealthy processed plant-based foods, including refined grains, sugary beverages, fried items, and highly processed plant-based products (such as plant-based meats laden with excessive sugar and salt, puffed snacks), despite being devoid of animal components, pose potential risks. Their high energy density, low nutritional value, and suspected links to metabolic dysregulation may contribute to an increased risk of chronic diseases [13]. Conversely, healthy plant-based foods, such as whole grains, fresh vegetables, fruits, legumes, nuts, and unrefined vegetable oils, are rich in beneficial components like dietary fiber, polyphenols, and unsaturated fatty acids [14,15]. Abundant evidence has confirmed their efficacy in reducing the risk of cardiovascular diseases, diabetes, and other ailments [14,16,17,18]. Rich in dietary fiber, vitamins, and minerals, Plant-based diets may offer protective effects against MetS [19,20]. A population-based study using nationally representative NHANES 2015–2016 data revealed that each additional serving of healthy plant-based foods was associated with a 4% lower risk of MetS, with the most pronounced protective effect observed among adults over 60 years of age [21]. Similarly, a intervention study in individuals with MetS demonstrated that participants assigned to follow a plant-based diet for 13 weeks exhibited significant improvements: dietary record analysis showed that higher adherence to a healthy plant-based dietary pattern was significantly correlated with reduced body weight and increased high-density lipoprotein (HDL) cholesterol levels [22]. Collectively, these findings suggest that a healthy plant-based diet is inversely associated with MetS risk, whereas an unhealthy plant-based dietary pattern may conversely elevate this risk [23,24,25].

Although nationwide studies consistently associate plant-based diets with reduced MetS risk [23,26], these findings may overlook regional dietary heterogeneity in China. Zhejiang Province exemplifies an Eastern healthy dietary pattern rich in vegetables [27], yet economic transitions have increased refined grains, ultra-processed foods, and take away consumption [28]. These regional dietary variations, rarely addressed in national studies, represent a significant literature gap. Existing research has predominantly focused on general associations at the national level, leaving the influence of regional dietary patterns on the relationship between plant-based diets and MetS largely unexplored. This cross-sectional study aims to fill this gap by evaluating PDI, hPDI, and uPDI, thereby providing evidence for context-specific, precision nutrition-based dietary guidelines to prevent MetS in Zhejiang and similar regions.

## 2. Materials and Methods

### 2.1. Study Design and Study Population

**Figure 1:**
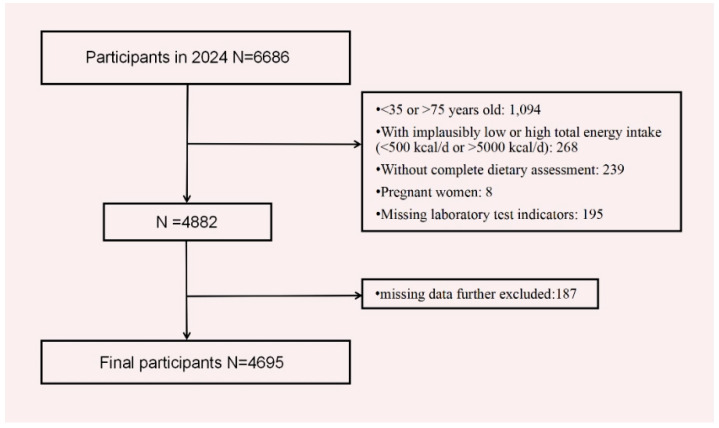
Flow chart of the analysis sample in this study.

All data used in this study were derived from the 2024 Zhejiang Province Residents Nutrition and Health Survey (ZJNHS). This survey employed a multi-stage stratified cluster random sampling method, selecting residents who have lived in Zhejiang Province for more than six months as the representative study population. Our study included detailed surveys in 33 districts/counties. For each monitoring site, two streets/towns were chosen, and for each street/town, two communities/villages were selected. The inclusion criteria for this study were as follows: participants must be aged 35–75 years, non-pregnant, and free from implausibly low or high total energy intake (defined as <500 kcal/day or >5000 kcal/day). Additionally, they were required to have available laboratory biomarker data necessary for the diagnosis of MetS and complete relevant baseline data. Individuals meeting all of these criteria were included in the study, while those failing to satisfy any of the above conditions were excluded. Informed consent was obtained from all participants. This study was approved by the Ethics Review Committee of Zhejiang Provincial Center for Disease Control and Prevention (Approval No.: 2024-019-01, 27 May 2024) (Figure 1).

### 2.2. Data Collection

A structured questionnaire was used to collect information from participants, including demographic characteristics (age, gender, ethnicity, and education level), lifestyle factors (total energy intake, physical activity, takeaway food consumption, smoking, and alcohol consumption), and health status (history of diabetes and hypertension). During the baseline survey, dietary assessments in the ZJNHS were conducted by trained professionals using a three 24 h dietary recall method. Participants were asked to report all foods consumed over three consecutive days, including meals eaten at home and away (two weekdays and one weekend day). We employed a weighing method to record the consumption of various cooking oils, salt, and other condiments over the same three-day period.

Physical examinations included measurements of height, weight, waist circumference, and blood pressure. Height was measured using the SZG-210 stadiometer (Yi lian, China), while weight was measured with the G&GTC-200K electronic scale (Shuang jie, China), allowing for the calculation of body mass index (BMI, kg/m^2^). Waist circumference was measured using a standardized method with a tape measure that was 1 cm wide and had a minimum scale of 0.1 cm. Blood pressure was measured using an Omron HBP1320 electronic sphygmomanometer(Omron, Kyoto, Japan), with measurements taken in the morning on the right arm (or the left arm if the right was not accessible). Participants were required to rest quietly for 5 min before measurement, with intervals of 1–2 min between each of the three measurements, and the average value was recorded. Laboratory tests included venous blood samples collected after an 8–12 h fast, with primary indicators including total cholesterol (TC), triglycerides (TG), high-density lipoprotein cholesterol (HDL-C), low-density lipoprotein cholesterol (LDL-C), fasting plasma glucose (FPG), and glycated hemoglobin (HbA1c). HDL-C was measured by the direct method, FPG by the hexokinase method, and TG by the free glycerol removal method.

### 2.3. Assessment of Plant-Based Diet Indices

We calculated the overall Plant-based diet index (PDI), healthy plant-based diet index (hPDI), and unhealthy plant-based diet index (uPDI) using the scoring criteria proposed by Satija et al. [11], with slight modifications to account for dietary habits in both Western and Chinese populations. Dietary data were collected using a hybrid method: a 3-day 24 h dietary recall, where participants self-reported intake under the guidance of trained investigators, combined with household weighing records for cooking oils, salt, and condiments. This approach integrated self-reported information for main meals and snacks with objective measurements for condiments to minimize recall bias. Specifically, we removed “potatoes,” added “tubers” (a category including yams and taro), and classified potatoes as a healthy plant-based food, as the Chinese Dietary Guidelines group them with other tubers [14]. Furthermore, we investigated cooking methods, classifying fried foods as a distinct category.

All reported foods were divided into 18 dietary groups: 8 healthy plant-based foods (whole grains/pulses, fruits, vegetables, nuts, soybeans and soy products, tubers, vegetable oils, tea, and coffee), 4 unhealthy plant-based foods (refined grains, sweets and sugar-sweetened beverages, preserved foods, and fried foods), and 6 animal-based foods (animal fats, milk and dairy products, eggs, fish and aquatic products, poultry meat, and red meat). The average daily intake of food items within each of the 18 categories was summed to calculate quintiles for each food group. Based on the emphasis of different food indices, food intake was assigned positive or negative scores. Positive scores were assigned by grouping the average daily intake of food categories into quintiles, with values assigned from 1 to 5 in ascending order. Negative scores were assigned similarly, but in reverse order, from 5 to 1. For individuals who did not consume a particular food group, the positive score was set to 1, and the negative score to 5. The scores for the 18 food groups were summed for each individual to obtain three indices, with a theoretical range of 18 to 90. In our analysis, we treated these indices as categorical variables (quintiles) and continuous variables (Supplementary Figure S1, Table S1).

### 2.4. Definition of the Metabolic Syndrome

The diagnosis of MetS was determined according to the guidelines for preventing and controlling type 2 diabetes in China (2020 Edition) [29]. Individuals were diagnosed with MetS if they met three or more of the following criteria: (1) abdominal obesity, defined as a waist circumference ≥ 90 cm in men or ≥85 cm in women; (2) elevated blood pressure (BP) ≥ 130/85 mmHg, a physician’s diagnosis of hypertension, or the use of antihypertensive medication; (3) elevated serum TG concentration ≥ 1.70 mmol/L; (4) elevated FPG ≥ 6.1 mmol/L or blood glucose ≥ 7.8 mmol/L 2 h after a glucose load, a physician’s diagnosis of diabetes, or the use of glucose-lowering medication; (5) reduced fasting serum HDL-C < 1.04 mmol/L.

### 2.5. Assessment of Covariates

Education level was categorized into four groups: illiteracy, junior high school or below, high school or vocational college, and university or above. Physical activity was defined based on the World Health Organization (WHO) guidelines for physical activity and sedentary behavior [30]: (1) insufficient physical activity: total weekly activity time less than 150 min; and (2) sufficient physical activity: total weekly activity time of 150 min or more. Smoking habits were classified into three categories: never smokers, former smokers, and current smokers. Similarly, drinking habits were categorized as never drinkers, former drinkers, and current drinkers.

BMI was calculated as weight in kilograms divided by height in meters squared (kg/m^2^). BMI residuals were derived from a linear regression model with waist circumference as the dependent variable and BMI as the independent variable, representing the difference between observed waist circumference and the model-predicted waist circumference based on BMI. This term reflects the portion of waist circumference variation that could not be explained by BMI. The models were adjusted for the following covariates: sex, age, ethnicity, region, family history of diabetes, family history of hypertension, smoking status, drinking status, takeaway food consumption, physical activity, education level, total energy intake, and BMI residuals.

### 2.6. Statistical Analysis

Statistical analyses were performed using R version 4.3.2 and SPSS 30.0. Participants were stratified into quintiles (Q1–Q5) based on their overall plant-based diet index (PDI), healthful plant-based diet index (hPDI), and unhealthful plant-based diet index (uPDI) scores, with continuous variables expressed as mean ± standard deviation (SD) and categorical variables as frequencies (percentages). Between-group comparisons utilized analysis of variance (ANOVA), Kruskal–Wallis test, chi-square test, or Fisher’s exact test as appropriate for data characteristics. Independent *t*-tests compared intake of 18 plant-based dietary components between metabolic syndrome (MetS) and non-MetS groups. Multivariable logistic regression models examined associations between plant-based diets and MetS (including components) using three adjustment levels: Model 1 (unadjusted); Model 2 (age- and sex-adjusted); Model 3 (further adjusted for ethnicity, residence area, family history of diabetes/hypertension, smoking, alcohol consumption, takeaway food frequency, physical activity, education, total energy intake, and BMI residuals). The Cochran–Armitage trend test assessed dose–response relationships across quintiles, while stratified analyses by age, sex, and residence area evaluated potential effect modifications. *p* < 0.05 was considered statistically significant.

## 3. Results

### 3.1. Characteristics of Participants

This study enrolled a total of 4695 participants, including 2551 females (54.3%) and 2144 males (45.7%). Among them, participants aged 55 years and above accounted for 55.8% (Table 1). In terms of health-related characteristics, females were more prevalent in the highest quintiles of PDI, uPDI, and hPDI, with the highest proportion observed in the hPDI quintile. Participants in the highest quintiles of PDI and uPDI were more likely to be aged 65–75 years, reside in rural areas, have an education level of middle school or below, and exhibit higher rates of smoking and alcohol consumption. However, the proportion of individuals engaging in sufficient physical activity was the lowest in the highest uPDI quintile. In contrast, participants in the highest hPDI quintile had lower rates of smoking and alcohol consumption, higher levels of physical activity, and a lower prevalence of obesity. Additionally, compared to the highest hPDI quintile, the highest quintiles of PDI and uPDI showed higher prevalence rates of abdominal obesity, hypertriglyceridemia, low HDL-C, elevated fasting glucose, and elevated blood pressure. Regarding nutrient intake, participants in the highest PDI quintile had higher total energy, fat, and carbohydrate intake, though no significant trend was observed for protein. In the highest hPDI quintile, total energy, protein, and fat intake generally decreased, while carbohydrate intake showed a gradual increase. Participants in the highest uPDI quintile had lower total protein intake, with total energy and fat intake also exhibiting a declining trend.

### 3.2. Food Characteristics

MetS patients demonstrated significantly lower consumption of whole grains/pulses (mean intake: 13.17 ± 27.13 g/d vs. 16.28 ± 31.97 g/d in non-MetS individuals; *p* < 0.05) and milk/dairy products (33.34 ± 79.67 g/d vs. 39.24 ± 77.59 g/d in non-MetS individuals; *p* < 0.05), but higher intake of vegetable oils (27.51 ± 18.97 g/d vs. 26.04 ± 18.35 g/d in non-MetS individuals; *p* < 0.05) and preserved foods (e.g., pickled vegetables, 6.94 ± 17.13 g/d vs. 5.02 ± 12.52 g/d in non-MetS individuals; *p* < 0.001) compared to non-MetS individuals (Supplementary Table S2 and Figure S1).

### 3.3. Association Between Plant-Based Diet Indices and MetS

In this sample, 23.9% of the overall population had MetS. Initial analysis adjusted for sex and age showed a 33% increased risk of MetS in the highest uPDI quintile. This positive association remained significant after additional adjustment for sociodemographic factors, health behaviors, total energy intake, and BMI residuals, with the highest quintile exhibiting a 37% elevated risk compared to the lowest (OR = 1.37, 95% CI: 1.08–1.73 *p*-trend = 0.013) (Table 2). In contrast, neither PDI nor hPDI showed significant associations with MetS risk (Supplementary Table S3).

Analysis of individual MetS components demonstrated significant associations between the highest uPDI quintile and adverse outcomes. The risk of abdominal obesity rose by 27% (OR = 1.27, 95% CI 1.01–1.59; *p*-trend = 0.032). For elevated fasting glucose, the odds increased by 35% (OR 1.35, 95% CI 1.06–1.72; *p*-trend = 0.017). The greatest effect was seen for elevated blood pressure, with a 43% higher risk (OR = 1.43, 95% CI 1.16–1.75, *p*-trend = 0.003) (Table 3).

### 3.4. Subgroup Analysis

**Figure 2:**
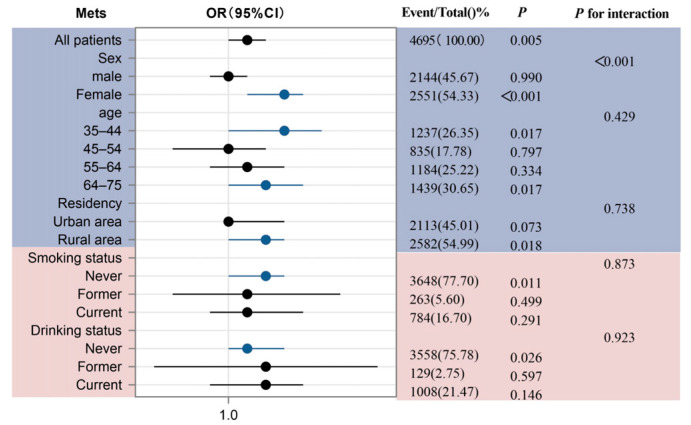
Subgroup analysis for the association between uPDI and MetS. The purple section represents basic demographic characteristics, including sex, age, and residency; The pink section represents health–related behavioral characteristics, covering smoking status and drinking status.

As illustrated in Figure 2, we stratified the analysis by sex, age, residency, smoking status, and alcohol consumption to evaluate potential effect modification in the association between uPDI and MetS. A significant interaction was observed for sex (*p* for interaction < 0.001), with a particularly strong association in females (OR 1.03, 95% CI 1.01–1.04; *p* < 0.001). No significant interactions were detected for other subgroups (all *p* for interaction > 0).

## 4. Discussion

To our knowledge, no prior studies have examined the associations of plant-based diets (assessed by PDI, hPDI, uPDI) with MetS and its components in adult populations of Zhejiang Province. This study found that higher adherence to the uPDI was associated with a 37% increased risk of MetS in the population of Zhejiang Province, China. Furthermore, the uPDI was significantly associated with three components of MetS: a 65% increased risk of elevated blood pressure, a 48% increased risk of elevated blood glucose, and a 31% increased risk of central obesity. These associations remained statistically significant after adjusting for covariates. However, the results for PDI and hPDI were not statistically significant. Notably, an interaction effect between uPDI and gender was observed, suggesting potential gender-specific differences in the relationship between plant-based diets and metabolic disease risk.

Multiple studies have validated the association of uPDI with both MetS and its components [14,31,32,33]. Two Chinese nationwide cohort studies provided important yet incomplete evidence. Huo’s team reported a 36% elevated abdominal obesity risk in the highest uPDI quartile without demonstrating significant association with MetS, whereas Chen’s group documented obesity risk reduction through hPDI but no protective effect against MetS [23,26]. Our study identified a distinct 37% higher metabolic syndrome risk linked to uPDI in Zhejiang Province. This regional discrepancy may be attributed to Zhejiang’s characteristic dietary pattern, characterized by high vegetable consumption and substantial intake of rice-based staples (e.g., white rice and processed rice products), supplemented by refined grain products such as refined noodles, bread, and refined rice flour [34,35]. Such a specific dietary combination may be obscured in nationwide studies encompassing diverse dietary cultures, underscoring the necessity of region-specific dietary guidelines.

Furthermore, both the French meta-analysis and Korean cohort demonstrated that uPDI elevates MetS and component risks more prominently in women [13,36]. Our study advances this evidence by confirming a significant gender interaction (*p* for interaction < 0.001) in Chinese populations, with stratified analyses revealing a stronger association in females (OR = 1.03, 95% CI 1.01–1.04; *p* < 0.001) than in males (OR = 1.00, 95% CI 0.99–1.01; *p* = 0.990). This disparity may stem from hormone-mediated lipid regulation and sex-specific dietary preferences, which could further exacerbate abdominal obesity and elevate cardiovascular risks in women [37,38]. Notably, unhealthy plant-based diet detrimental effects extend beyond metabolic health, with emerging links to depression and anxiety [39], underscoring diet quality’s multidimensional impact. This phenomenon aligns with research showing that emotional eating acts as a coping strategy for negative emotions. Chinese young adult females are more susceptible to emotional eating than males are, especially those who display a marked preference for high-fat foods during stressful scenarios, which is indicative of a stress-driven high-fat dietary pattern [40,41]. Meanwhile, external food cues such as flavor-focused packaging disproportionately suppress women’s attention to nutritional risk information. Research indicates that women’s attention to nutritional information is more readily impaired by food packaging and flavor labels, particularly showing the greatest decline in attention for desserts, puffed snacks, frozen convenience foods, and sauces [42]. These findings collectively highlight the urgency of optimizing dietary patterns for MetS prevention, while future studies should explore how regional dietary variations modulate these gender-specific associations.

Moreover, our study observed significant associations between the uPDI and MetS components (e.g., hyperglycemia, central obesity, and hypertension). Although the cross-sectional design precludes direct causal inference, existing literature supports potential mechanistic explanations. First, the high sugar and refined grain content in unhealthy plant-based diets may contribute to insulin resistance and hyperglycemia [43,44], while high-fat and high-sugar components could promote central obesity by disrupting lipid metabolism and adipocyte function [45]. Additionally, high salt and fat intake may exacerbate hypertension risk by affecting vascular function and the renin-angiotensin system [46,47]. These findings suggest that unhealthy plant-based diets may collectively elevate MetS risk through multiple mechanisms, including insulin resistance, lipid metabolism dysregulation, and abnormal blood pressure regulation [48,49].

Our study found no significant associations between PDI/hPDI and MetS, contrasting with previous studies [7,50,51,52,53]. The discrepancy may stem from Zhejiang Province’s dietary patterns, characterized by higher overall plant-based food intake and Jiangnan cuisine features (e.g., Hangzhou-style dishes) that combine meat and vegetables rich in monounsaturated fatty acids (MUFA) but lack polyunsaturated fatty acids (PUFA) in purely plant-based dishes [54,55,56]. Notably, dietary analysis revealed the MetS group consumed significantly less whole grains/pulses (13.17 vs. 16.28 g/d, *p* < 0.001) but more vegetable oils (27.51 vs. 26.04 g/d, *p* = 0.020) than the non-MetS group, consistent with Zhejiang’s prevalent use of vegetable oils for cooking. The MetS group also showed higher preserved food (6.94 vs. 5.02 g/d, *p* < 0.001) and lower dairy intake (33.34 vs. 39.24 g/d, *p* = 0.030) compared to the non-MetS group. These findings suggest that in regions with high plant-based food consumption like Zhejiang, unhealthy plant-based foods (e.g., preserved foods) may exert amplified adverse effects, while the protective potential of healthy plant-based foods is attenuated due to insufficient intake. Consequently, the weaker associations between PDI/hPDI and MetS observed in this study, compared to Western or Northern Chinese populations, may reflect the reduced discriminative power of these indices for metabolic risk in the dietary context of the study’s population (e.g., Southern Chinese diets) [57,58].

A key strength of this study lies in its utilization of the latest 2024 data from Zhejiang Province, which reflects recent dietary transitions, when combined with a comprehensive assessment of plant-based diet quality. The sample size encompassed multiple regions within Zhejiang Province, enhancing the representativeness of the findings and their applicability to the provincial population. Additionally, adjusting for covariates using BMI residuals further improved the reliability of the results. However, this study has several limitations. The reliance on self-reported dietary data through the three-day 24 h recall method may introduce recall bias, potentially affecting the accuracy of dietary intake measurements. While the cross-sectional design can reveal associations, it cannot establish causality, and residual confounding factors may persist despite adjustments for multiple covariates.

## 5. Conclusions

Current findings demonstrate that an unhealthy plant-based diet is significantly associated with an increased risk of MetS, including hypertension, hyperglycemia, and central obesity, with stronger associations observed in women. These results suggest that future dietary interventions should consider sex-specific approaches. Subsequent studies should conduct multi-center longitudinal investigations. More precise nutrition intervention strategies should be implemented in specific populations.

## Figures and Tables

**Table 1:** Baseline characteristic of ZJNHS according to quintiles of plant-based diet indices (*n* = 4695).

Characteristic	Total	Q1	Q2	Q3	Q4	Q5	*p*
PDI							
Sample size, n	4695	897	976	836	985	1001	
Ethnic group, n (%)							0.307
Han	4663 (99.3)	889 (99.1)	970 (99.4)	827 (98.9)	979 (99.4)	998 (99.7)	
Others	32 (0.7)	8 (0.9)	6 (0.6)	9 (1.1)	6 (0.6)	3 (0.3)	
Sex, n (%)							0.704
Male	2144 (45.7)	396 (44.2)	448 (45.9)	385 (46)	442 (44.9)	473 (47.2)	
Female	2551 (54.3)	501 (55.8)	528 (54.1)	451 (54)	543 (55.1)	528 (52.8)	
Age, year							0.002
35–44	1237 (26.4)	259 (28.9)	265 (27.2)	233 (27.9)	262 (26.6)	218 (21.8)	
45–54	835 (17.8)	168 (18.7)	191 (19.6)	149 (17.8)	166 (16.8)	161 (16.1)	
55–64	1184 (25.2)	224 (25)	236 (24.2)	215 (25.7)	246 (25)	263 (26.3)	
64–75	1439 (30.6)	246 (27.4)	284 (29.1)	239 (28.6)	311 (31.6)	359 (35.9)	
Residency, n (%)							0.038
Urban area	2113 (45)	407 (45.4)	409 (41.9)	360 (43.1)	477 (48.4)	460 (46)	
Rural area	2582 (55)	490 (54.6)	567 (58.1)	476 (56.9)	508 (51.6)	541 (54)	
Education, year							<0.001
Illiteracy	325 (6.9)	66 (7.4)	68 (7)	72 (8.6)	73 (7.4)	46 (4.6)	
Middle school and below	2659 (56.6)	485 (54.1)	535 (54.8)	449 (53.7)	557 (56.6)	633 (63.2)	
Secondary schools and junior Colleges	1070 (22.8)	190 (21.2)	239 (24.5)	203 (24.3)	223 (22.6)	215 (21.5)	
University and above	641 (13.6)	156 (17.4)	134 (13.7)	112 (13.4)	132 (13.4)	107 (10.7)	
Physical activity, n (%)							<0.001
Insufficient	2046 (43.6)	447 (49.8)	426 (43.6)	337 (40.3)	443 (45)	393 (39.3)	
Sufficient	2649 (56.4)	450 (50.2)	550 (56.4)	499 (59.7)	542 (55)	608 (60.7)	
Smoking status, n (%)							0.155
Never	3648 (77.7)	726 (80.9)	771 (79)	646 (77.3)	741 (75.2)	764 (76.3)	
Former	263 (5.6)	41 (4.6)	54 (5.5)	47 (5.6)	65 (6.6)	56 (5.6)	
Current	784 (16.7)	130 (14.5)	151 (15.5)	143 (17.1)	179 (18.2)	181 (18.1)	
Drinking status, n (%)							0.002
Never	3558 (75.8)	721 (80.4)	743 (76.1)	612 (73.2)	735 (74.6)	747 (74.6)	
Former	129 (2.8)	13 (1.4)	18 (1.8)	27 (3.2)	37 (3.8)	34 (3.4)	
Current	1008 (21.5)	163 (18.2)	215 (22)	197 (23.6)	213 (21.6)	220 (22)	
Takeaway, n (times/week)							0.201
≤2	4406 (93.8)	845 (94.2)	917 (94)	777 (92.9)	925 (93.9)	942 (94.1)	
3–6	248 (5.3)	46 (5.1)	49 (5)	54 (6.5)	49 (5)	50 (5)	
7–10	25 (0.5)	2 (0.2)	3 (0.3)	3 (0.4)	9 (0.9)	8 (0.8)	
>10	16 (0.3)	4 (0.4)	7 (0.7)	2 (0.2)	2 (0.2)	1 (0.1)	
Family history							
Diabetes, n (%)							0.397
No	3918 (83.4)	754 (84.1)	808 (82.8)	706 (84.4)	832 (84.5)	818 (81.7)	
Yes	777 (16.6)	143 (15.9)	168 (17.2)	130 (15.6)	153 (15.5)	183 (18.3)	
Hypertension, n (%)							0.019
No	2825 (60.2)	554 (61.8)	618 (63.4)	508 (60.8)	580 (58.9)	565 (56.4)	
Yes	1869 (39.8)	343 (38.2)	357 (36.6)	328 (39.2)	405 (41.1)	436 (43.6)	
BMI, n (kg/m^2^)							0.569
Lean (<18.5)	163 (3.5)	30 (3.3)	32 (3.3)	21 (2.5)	43 (4.4)	37 (3.7)	
Normal (18.5~23.9)	2318 (49.4)	440 (49)	479 (49.1)	420 (50.2)	494 (50.2)	485 (48.4)	
Overweight (24~27.9)	1695 (36.1)	314 (35)	349 (35.8)	310 (37.1)	348 (35.3)	374 (37.4)	
Obesity (≥28)	519 (11)	113 (12.6)	116 (11.9)	85 (10.2)	100 (10.2)	105 (10.5)	
WC, (cm)	83.9 ± 10.0	83.2 ± 10.6	84.1 ± 10.2	83.9 ± 9.8	83.9 ± 9.8	84.1 ± 9.8	0.29
SBP, mmHg	126.1 ± 15.4	125.6 ± 15.7	125.0 ± 14.5	126.5 ± 15.6	126.0 ± 15.9	127.4 ± 15.4	0.011
DBP, mmHg	77.7 ± 9.3	77.5 ± 9.0	77.2 ± 8.7	77.86 ± 9.4	77.7 ± 9.6	78.2 ± 9.7	0.131
Abdominal obesity, n (%)							0.575
No	3035 (64.6)	598 (66.7)	632 (64.8)	541 (64.7)	620 (62.9)	644 (64.3)	
Yes	1660 (35.4)	299 (33.3)	344 (35.2)	295 (35.3)	365 (37.1)	357 (35.7)	
High fasting glucose, n (%)							0.059
No	3655 (77.9)	708 (78.9)	775 (79.4)	668 (79.9)	748 (76)	756 (75.5)	
Yes	1039 (22.1)	189 (21.1)	201 (20.6)	168 (20.1)	236 (24)	245 (24.5)	
Elevated blood pressure, n (%)							0.089
No	2678 (57)	514 (57.3)	576 (59)	483 (57.8)	572 (58.1)	533 (53.2)	
Yes	2017 (43)	383 (42.7)	400 (41)	353 (42.2)	413 (41.9)	468 (46.8)	
Hypertriglyceridemia, n (%)							0.577
No	2981 (63.5)	578 (64.4)	608 (62.3)	547 (65.4)	624 (63.4)	624 (62.3)	
Yes	1714 (36.5)	319 (35.6)	368 (37.7)	289 (34.6)	361 (36.6)	377 (37.7)	
Low HDL-C, n (%)							0.701
No	3837 (81.7)	741 (82.6)	803 (82.3)	689 (82.4)	797 (80.9)	807 (80.6)	
Yes	858 (18.3)	156 (17.4)	173 (17.7)	147 (17.6)	188 (19.1)	194 (19.4)	
MetS, n (%)							0.826
No	3573 (76.1)	684 (76.2)	750 (76.8)	641 (76.7)	750 (76.1)	748 (74.7)	
Yes	1122 (23.9)	213 (23.8)	226 (23.2)	195 (23.3)	235 (23.9)	253 (25.3)	
Healthy plant foods (g/day)	371.6 ± 200.6	284.3 ± 146.8	336.0 ± 200.4	375.5 ± 194.9	394.8 ± 185.8	458.5 ± 220.4	<0.001
Less-healthy plant foods (g/day)	224.1 ± 105.7	184.4 ± 82.4	211.3 ± 97.1	223.9 ± 97.4	234.0 ± 100.9	262.7 ± 126.8	<0.001
Animal Total, Mean ± SD	274.3 ± 139.0	339.3 ± 136.4	294.9 ± 140.3	280.9 ± 148.2	249.4 ± 119.6	214.8 ± 118.2	<0.001
Total engery, kcal	1691.9 ± 446.4	1569.2 ± 398.0	1662.4 ± 441.0	1717.2 ± 449.8	1714.8 ± 446.2	1787.1 ± 463.0	<0.001
Total Protein, g	70.1 ± 22.7	70.6 ± 21.9	70.7 ± 23.3	71.6 ± 24.3	68.9 ± 21.6	68.7 ± 22.7	0.024
Total Fat, g	69.8 ± 27.8	68.1 ± 27.2	69.8 ± 27.9	70.9 ± 28.1	69.2 ± 27.2	71.2 ± 28.4	0.094
Total Carbohydrate, g	199.6 ± 67.5	172.3 ± 60.3	191.7 ± 67.9	201.3 ± 67.6	208.0 ± 65.9	222.1 ± 65.0	<0.001
hPDI							
Sample size, n	4695	889	764	908	1114	1020	
Ethnic group, n (%)							0.207
Han	4663 (99.3)	887 (99.8)	756 (99.0)	904 (99.6)	1105 (99.2)	1011 (99.1)	
Others	32 (0.7)	2 (0.2)	8 (1.0)	4 (0.4)	9 (0.8)	9 (0.9)	
Sex, n (%)							0.012
Male	2144 (45.7)	435 (48.9)	363 (47.5)	408 (44.9)	516 (46.3)	422 (41.4)	
Female	2551 (54.3)	454 (51.1)	401 (52.5)	500 (55.1)	598 (53.7)	598 (58.6)	
Age, year							<0.001
35–44	1237 (26.4)	261 (29.4)	225 (29.4)	235 (25.9)	295 (26.5)	221 (21.7)	
45–54	835 (17.8)	174 (19.6)	141 (18.5)	172 (18.9)	186 (16.7)	162 (15.9)	
55–64	1184 (25.2)	200 (22.5)	171 (22.4)	218 (24)	294 (26.4)	301 (29.5)	
64–75	1439 (30.6)	254 (28.6)	227 (29.7)	283 (31.2)	339 (30.4)	336 (32.9)	
Residency, n (%)							<0.001
Urban area	2113 (45)	383 (43.1)	373 (48.8)	395 (43.5)	444 (39.9)	518 (50.8)	
Rural area	2582 (55)	506 (56.9)	391 (51.2)	513 (56.5)	670 (60.1)	502 (49.2)	
Education, year							0.001
Illiteracy	325 (6.9)	71 (8)	44 (5.8)	62 (6.8)	83 (7.4)	65 (6.4)	
Middle school and below	2659 (56.6)	466 (52.4)	401 (52.5)	515 (56.7)	670 (60.1)	607 (59.5)	
Secondary schools and junior Colleges	1070 (22.8)	210 (23.6)	191 (25)	217 (23.9)	228 (20.5)	224 (22)	
University and above	641 (13.6)	142 (16)	128 (16.8)	114 (12.6)	133 (11.9)	124 (12.2)	
Physical activity, n (%)							0.05
Insufficient	2046 (43.6)	401 (45.1)	356 (46.6)	405 (44.6)	475 (42.6)	409 (40.1)	
Sufficient	2649 (56.4)	488 (54.9)	408 (53.4)	503 (55.4)	639 (57.4)	611 (59.9)	
Smoking status, n (%)							0.056
Never	3648 (77.7)	672 (75.6)	594 (77.8)	710 (78.2)	849 (76.2)	823 (80.7)	
Former	263 (5.6)	49 (5.5)	43 (5.6)	51 (5.6)	58 (5.2)	62 (6.1)	
Current	784 (16.7)	168 (18.9)	127 (16.6)	147 (16.2)	207 (18.6)	135 (13.2)	
Drinking status, n (%)							0.667
Never	3558 (75.8)	659 (74.1)	585 (76.6)	682 (75.1)	842 (75.6)	790 (77.4)	
Former	129 (2.8)	21 (2.4)	25 (3.3)	25 (2.8)	32 (2.9)	26 (2.6)	
Current	1008 (21.5)	209 (23.5)	154 (20.2)	201 (22.1)	240 (21.5)	204 (20)	
Takeaway, n (times/week)							0.043
≤2	4406 (93.8)	838 (94.3)	699 (91.5)	848 (93.4)	1051 (94.3)	970 (95.1)	
3–6	248 (5.3)	44 (5)	56 (7.3)	50 (5.5)	56 (5)	42 (4.1)	
7–10	25 (0.5)	1 (0.1)	7 (0.9)	6 (0.7)	4 (0.4)	7 (0.7)	
>10	16 (0.3)	6 (0.7)	2 (0.3)	4 (0.4)	3 (0.3)	1 (0.1)	
Family history							
Diabetes, n (%)							0.701
No	3918 (83.4)	747 (84)	641 (83.9)	767 (84.5)	923 (82.8)	840 (82.4)	
Yes	777 (16.6)	142 (16)	123 (16.1)	141 (15.5)	191 (17.2)	180 (17.6)	
Hypertension, n (%)							0.338
No	2825 (60.2)	519 (58.4)	463 (60.6)	571 (63)	667 (59.9)	605 (59.3)	
Yes	1869 (39.8)	370 (41.6)	301 (39.4)	336 (37)	447 (40.1)	415 (40.7)	
BMI, n (kg/m^2^)							0.548
Lean (<18.5)	163 (3.5)	32 (3.6)	29 (3.8)	29 (3.2)	37 (3.3)	36 (3.5)	
Normal (18.5~23.9)	2318 (49.4)	426 (47.9)	387 (50.6)	430 (47.4)	552 (49.6)	523 (51.3)	
Overweight (24~27.9)	1695 (36.1)	323 (36.3)	263 (34.4)	339 (37.3)	398 (35.7)	372 (36.5)	
Obesity (≥28)	519 (11)	108 (12.2)	85 (11.1)	110 (12.1)	127 (11.4)	89 (8.7)	
WC, (cm)	83.9 ± 10.0	83.9 ± 10.1	83.9 ± 10.3	84.3 ± 10.0	84.0 ± 10.1	83.3 ± 9.7	0.221
SBP, mmHg	126.1 ± 15.4	126.4 ± 14.5	125.5 ± 14.9	125.6 ± 15.8	126.3 ± 15.7	126.5 ± 16.0	0.556
DBP, mmHg	77.7 ± 9.3	77.9 ± 8.9	77.4 ± 9.0	77.5 ± 9.4	77.9 ± 9.5	77.6 ± 9.5	0.787
Abdominal obesity, n (%)							0.582
No	3035 (64.6)	565 (63.6)	507 (66.4)	574 (63.2)	718 (64.4)	671 (65.8)	
Yes	1660 (35.4)	324 (36.4)	257 (33.6)	334 (36.8)	396 (35.6)	349 (34.2)	
High fasting glucose, n (%)							0.455
No	3655 (77.9)	705 (79.3)	603 (78.9)	709 (78.2)	862 (77.4)	776 (76.1)	
Yes	1039 (22.1)	184 (20.7)	161 (21.1)	198 (21.8)	252 (22.6)	244 (23.9)	
Elevated blood pressure, n (%)							0.689
No	2678 (57)	505 (56.8)	454 (59.4)	517 (56.9)	626 (56.2)	576 (56.5)	
Yes	2017 (43)	384 (43.2)	310 (40.6)	391 (43.1	488 (43.8)	444 (43.5)	
Hypertriglyceridemia, n (%)							0.387
No	2981 (63.5)	560 (63)	504 (66)	584 (64.3)	686 (61.6)	647 (63.4)	
Yes	1714 (36.5)	329 (37)	260 (34)	324 (35.7)	428 (38.4)	373 (36.6)	
Low HDL-C, n (%)							0.969
No	3837 (81.7)	727 (81.8)	628 (82.2)	743 (81.8)	913 (82)	826 (81)	
Yes	858 (18.3)	162 (18.2)	136 (17.8)	165 (18.2)	201 (18)	194 (19)	
MetS, n (%)							0.948
No	3573 (76.1)	671 (75.5)	582 (76.2)	685 (75.4)	853 (76.6)	782 (76.7)	
Yes	1122 (23.9)	218 (24.5)	182 (23.8)	223 (24.6)	261 (23.4)	238 (23.3)	
Healthy plant foods (g/day)	371.6 ± 200.6	293.5 ± 179.7	347.9 ± 202.0	361.9 ± 200.9	388.4 ± 193.2	447.7 ± 195.2	<0.001
Less-healthy plant foods (g/day)	224.1 ± 105.7	245.3 ± 101.6	230.0 ± 99.0	229.4 ± 111.8	217.7 ± 109.9	203.4 ± 99.5	<0.001
Animal Total, Mean ± SD	274.3 ± 139.0	325.6 ± 137.3	304.7 ± 135.4	279.7 ± 137.7	252.5 ± 126.9	225.6 ± 135.7	<0.001
Total engery, kcal	1691.9 ± 446.4	1702.1 ± 433.7	1699.5 ± 419.1	1696.3 ± 451.9	1672.3 ± 457.5	1694.9 ± 459.8	0.566
Total Protein, g	70.1 ± 22.7	75.7 ± 22.7	72.5 ± 21.1	70.5 ± 22.5	67.1 ± 22.3	66.2 ± 23.5	<0.001
Total Fat, g	69.8 ± 27.8	68.8 ± 27.7	71.1 ± 28.5	70.7 ± 28.1	69.2 ± 28.4	69.6 ± 26.3	0.359
Total Carbohydrate, g	199.6 ± 67.5	197.5 ± 64.0	196.2 ± 65.2	199.2 ± 69.3	198.6 ± 66.3	205.4 ± 71.4	0.033
uPDI							
Sample size, n	4695	891	968	727	1100	1009	
Ethnic group, n (%)							0.8
Han	4663 (99.3)	883 (99.1)	960 (99.2)	723 (99.4)	1093 (99.4)	1004 (99.5)	
Others	32 (0.7)	8 (0.9)	8 (0.8)	4 (0.6)	7 (0.6)	5 (0.5)	
Sex, n (%)							0.939
Male	2144 (45.7)	409 (45.9)	447 (46.2)	329 (45.2)	509 (46.3)	450 (44.6)	
Female	2551 (54.3)	482 (54.1)	521 (53.8)	398 (54.8)	591 (53.7)	559 (55.4)	
Age, year							<0.001
35–44	1237 (26.4)	293 (32.9)	292 (30.2)	193 (26.6)	255 (23.2)	204 (20.2)	
45–54	835 (17.8)	179 (20.1)	155 (16)	141 (19.4)	185 (16.8)	175 (17.3)	
55–64	1184 (25.2)	208 (23.3)	232 (24)	174 (23.9)	313 (28.4)	257 (25.5)	
64–75	1439 (30.6)	211 (23.7)	289 (29.9)	219 (30.1)	347 (31.6)	373 (37)	
Residency, n (%)							<0.001
Urban area	2113 (45)	578 (64.9)	508 (52.5)	293 (40.3)	431 (39.2)	303 (30)	
Rural area	2582 (55)	313 (35.1)	460 (47.5)	434 (59.7)	669 (60.8)	706 (70)	
Education, year							<0.001
Illiteracy	325 (6.9)	32 (3.6)	56 (5.8)	67 (9.2)	82 (7.4)	88 (8.7)	
Middle school and below	2659 (56.6)	412 (46.2)	516 (53.3)	399 (54.9)	657 (59.7)	675 (66.9)	
Secondary schools and junior colleges	1070 (22.8)	260 (29.2)	247 (25.5)	166 (22.8)	226 (20.6)	171 (17)	
University and above	641 (13.6)	187 (21)	149 (15.4)	95 (13.1)	135 (12.3)	75 (7.4)	
Physical activity, n (%)							0.037
Insufficient	2046 (43.6)	362 (40.6)	427 (44.1)	310 (42.6)	468 (42.6)	479 (47.5)	
Sufficient	2649 (56.4)	529 (59.4)	541 (55.9)	417 (57.4)	632 (57.4)	530 (52.5)	
Smoking status, n (%)							0.018
Never	3648 (77.7)	714 (80.1)	771 (79.6)	553 (76.1)	837 (76.1)	773 (76.6)	
Former	263 (5.6)	51 (5.7)	49 (5.1)	34 (4.7)	79 (7.2)	50 (5)	
Current	784 (16.7)	126 (14.1)	148 (15.3)	140 (19.3)	184 (16.7)	186 (18.4)	
Drinking status, n (%)							0.229
Never	3558 (75.8)	689 (77.3)	740 (76.4)	565 (77.7)	822 (74.7)	742 (73.5)	
Former	129 (2.8)	19 (2.1)	31 (3.2)	14 (1.9)	37 (3.4)	28 (2.8)	
Current	1008 (21.5)	183 (20.5)	197 (20.4)	148 (20.4)	241 (21.9)	239 (23.7)	
Takeaway, n (times/week)							<0.001
≤2	4406 (93.8)	813 (91.2)	902 (93.2)	671 (92.3)	1046 (95.1)	974 (96.5)	
3–6	248 (5.3)	67 (7.5)	60 (6.2)	44 (6)	48 (4.4)	29 (2.9)	
7–10	25 (0.5)	8 (0.9)	3 (0.3)	6 (0.8)	5 (0.4)	3 (0.3)	
>10	16 (0.3)	3 (0.3)	3 (0.3)	6 (0.8)	1 (0.1)	3 (0.3)	
Family history							
Diabetes, n (%)							0.09
No	3918 (83.4)	717 (80.5)	808 (83.5)	609 (83.8)	926 (84.2)	858 (85)	
Yes	777 (16.6)	174 (19.5)	160 (16.5)	118 (16.2)	174 (15.8)	151 (15)	
Hypertension, n (%)							0.033
No	2825 (60.2)	499 (56)	578 (59.7)	436 (60.1)	678 (61.6)	634 (62.8)	
Yes	1869 (39.8)	392 (44)	390 (40.3)	290 (39.9)	422 (38.4)	375 (37.2)	
BMI, n (kg/m^2^)							0.349
Lean (<18.5)	163 (3.5)	31 (3.5)	28 (2.9)	31 (4.3)	38 (3.4)	35 (3.5)	
Normal (18.5~23.9)	2318 (49.4)	466 (52.3)	494 (51)	345 (47.5)	536 (48.7)	477 (47.3)	
Overweight (24~27.9)	1695 (36.1)	313 (35.1)	349 (36)	261 (35.9)	400 (36.4)	372 (36.9)	
Obesity (≥28)	519 (11)	81 (9.1)	97 (10)	90 (12.4)	126 (11.4)	125 (12.4)	
WC, (cm)	83.87 ± 10.02	83.17 ± 9.94	83.36 ± 9.94	83.62 ± 10.66	84.26 ± 9.82	84.73 ± 9.85	0.003
SBP, mmHg	126.09 ± 15.44	123.68 ± 14.32	125.30 ± 15.21	126.35 ± 16.03	127.12 ± 16.21	127.68 ± 15.04	<0.001
DBP, mmHg	77.68 ± 9.29	76.41 ± 8.88	77.02 ± 8.84	78.42 ± 9.87	77.90 ± 9.34	78.68 ± 9.41	<0.001
Abdominal obesity, n (%)							0.011
No	3035 (64.6)	596 (66.9)	653 (67.5)	476 (65.5)	697 (63.4)	613 (60.8)	
Yes	1660 (35.4)	295 (33.1)	315 (32.5)	251 (34.5)	403 (36.6)	396 (39.2)	
High fasting glucose, n (%)							0.013
No	3655 (77.9)	725 (81.4)	756 (78.1)	570 (78.4)	851 (77.4)	753 (74.6)	
Yes	1039 (22.1)	166 (18.6)	212 (21.9)	157 (21.6)	248 (22.6)	256 (25.4)	
Elevated blood pressure, n (%)							<0.001
No	2678 (57)	582 (65.3)	548 (56.6)	412 (56.7)	598 (54.4)	538 (53.3)	
Yes	2017 (43)	309 (34.7)	420 (43.4)	315 (43.3)	502 (45.6)	471 (46.7)	
Hypertriglyceridemia, n (%)							0.443
No	2981 (63.5)	580 (65.1)	628 (64.9)	460 (63.3)	693 (63)	620 (61.4)	
Yes	1714 (36.5)	311 (34.9)	340 (35.1)	267 (36.7)	407 (37)	389 (38.6)	
Low HDL-C, n (%)							0.785
No	3837 (81.7)	715 (80.2)	791 (81.7)	598 (82.3)	903 (82.1)	830 (82.3)	
Yes	858 (18.3)	176 (19.8)	177 (18.3)	129 (17.7)	197 (17.9)	179 (17.7)	
MetS, n (%)							0.025
No	3573 (76.1)	705 (79.1)	745 (77)	558 (76.8)	830 (75.4)	735 (72.8)	
Yes	1122 (23.9)	186 (20.9)	223 (23)	169 (23.2)	270 (24.6)	274 (27.2)	
Healthy plant foods (g/day)	371.6 ± 200.6	491.3 ± 205.1	409.3 ± 181.7	362.3 ± 150.9	332.2 ± 190.7	279.5 ± 195.8	<0.001
Less-healthy plant foods (g/day)	224.1 ± 105.7	183.0 ± 74.3	207.1 ± 93.5	223.7 ± 109.0	234.7 ± 111.6	265.4 ± 114.2	<0.001
Animal Total, Mean ± SD	274.3 ± 139.0	379.6 ± 136.5	315.8 ± 128.1	263.2 ± 117.4	241.3 ± 116.3	185.3 ± 111.2	<0.001
Total engery, kcal	1691.9 ± 446.4	1809.3 ± 434.1	1735.3 ± 423.1	1684.1 ± 451.1	1655.0 ± 457.3	1592.5 ± 435.5	<0.001
Total Protein, g	70.1 ± 22.7	80.6 ± 22.3	74.5 ± 21.4	69.8 ± 21.4	66.8 ± 21.3	60.1 ± 21.9	<0.001
Total Fat, g	69.8 ± 27.8	78.7 ± 25.7	74.6 ± 26.8	69.8 ± 27.0	67.1 ± 28.0	60.5 ± 27.5	<0.001
Total Carbohydrate, g	199.6 ± 67.5	199.7 ± 61.5	195.8 ± 63.4	197.9 ± 67.5	198.8 ± 70.1	205.2 ± 72.8	0.031

Descriptive analyses of continuous variables were performed first, including calculations of means and standard deviations. For group comparisons, continuous variables were then compared using ANOVA (*F*-test); categorical variables were analyzed via the Chi-square test (*χ*^2^); Fisher’s exact test was applied when expected cell counts < 5. BMI, body mass index; WC, waist circumference; SBP, systolic blood pressure; DBP, diastolic blood pressure.

**Table 2:** Associations between uPDI and MetS (N = 4695).

	Q1	Q2	Q3	Q4	Q5	*p*-Trend
	uPDI					
Model 1	Ref	1.13 (0.91~1.41)	1.15 (0.91~1.45)	1.23 (1.00~1.52)	1.41 (1.14~1.75)	0.001
Model 2	Ref	1.10 (0.88~1.38)	1.12 (0.88~1.42)	1.16 (0.93~1.44)	1.33 (1.07~1.65)	0.01
Model 3	Ref	1.16 (0.92~1.46)	1.20 (0.93~1.54)	1.19 (0.95~1.49)	1.37 (1.08~1.73)	0.013

Model 1 was unadjusted; Model 2 adjusted for age and sex; and Model 3 further adjusted for ethnicity, region, family history of diabetes and hypertension, smoking status, alcohol status, takeaway food consumption, physical activity level, education level, total energy intake, and BMI residuals.

**Table 3:** Associations between unhealthy plant-based diet indices and MetS components (N = 4695).

	Q1	Q2	Q3	Q4	Q5	*p*-Trend
	uPDI					
Abdominal obesity	Ref	1.01 (0.81~1.26)	1.21 (0.95~1.54)	1.09 (0.87~1.35)	1.27 (1.01~1.59)	0.032
High fasting glucose	Ref	1.21 (0.95~1.54)	1.20 (0.92~1.56)	1.18 (0.93~1.49)	1.35 (1.06~1.72)	0.037
Elevated blood pressure	Ref	1.41 (1.16~1.73)	1.38 (1.11~1.72)	1.44 (1.18~1.75)	1.43 (1.16~1.75)	0.003
Hypertriglyceridemia	Ref	1.02 (0.84~1.24)	1.10 (0.89~1.35)	1.09 (0.90~1.32)	1.17 (0.95~1.42)	0.104
Low HDL-C	Ref	0.92 (0.73~1.18)	0.92 (0.70~1.19)	0.93 (0.73~1.18)	0.94 (0.73~1.21)	0.685

Model adjustments: Models were adjusted for age and sex; and further for ethnicity, region, family history of diabetes and hypertension, smoking status, alcohol status, takeaway food consumption, physical activity, education level, total energy intake, and BMI residuals. Low HDL-C: low high-density lipoprotein cholesterol.

## Data Availability

The data presented in this study are available on request from the corresponding author due to privacy and ethical concerns, as the data contain sensitive personal and health information of participants in the Zhejiang region. For specific inquiries, please contact the corresponding author: rhzhang@cdc.zj.cn.

## Supplementary Materials

The Supplementary Materials for this article can be found online at: https://www.mdpi.com/article/10.3390/nu17132159/s1. Figure S1: Characteristics of 18 Plant-Based Dietary Components in Metabolic Syndrome; Table S1: Plant-based Diet Indices Construction, Scoring, and Food Items; Table S2: Characteristics of 18 Plant-Based Dietary Components in Metabolic Syndrome; Table S3: Associations between PDI, hPDI, and MetS (*n* = 4695).
