# Supplementary material for: Association Between Plant-Based Diets and Metabolic Syndrome in Zhejiang, China: A Cross-Sectional Study
Source: Nutrients. 2025 Jun 28;17(13):2159. doi: 10.3390/nu17132159 (PMC12252280; doi:10.3390/nu17132159)
Supplement: Supplementary file 1 [file nutrients-17-02159-s001.zip › nutrients-3712821-supplementary.pdf]

Figure S1.Characteristics of 18 Plant-Based Dietary Components in Metabolic Syndrome

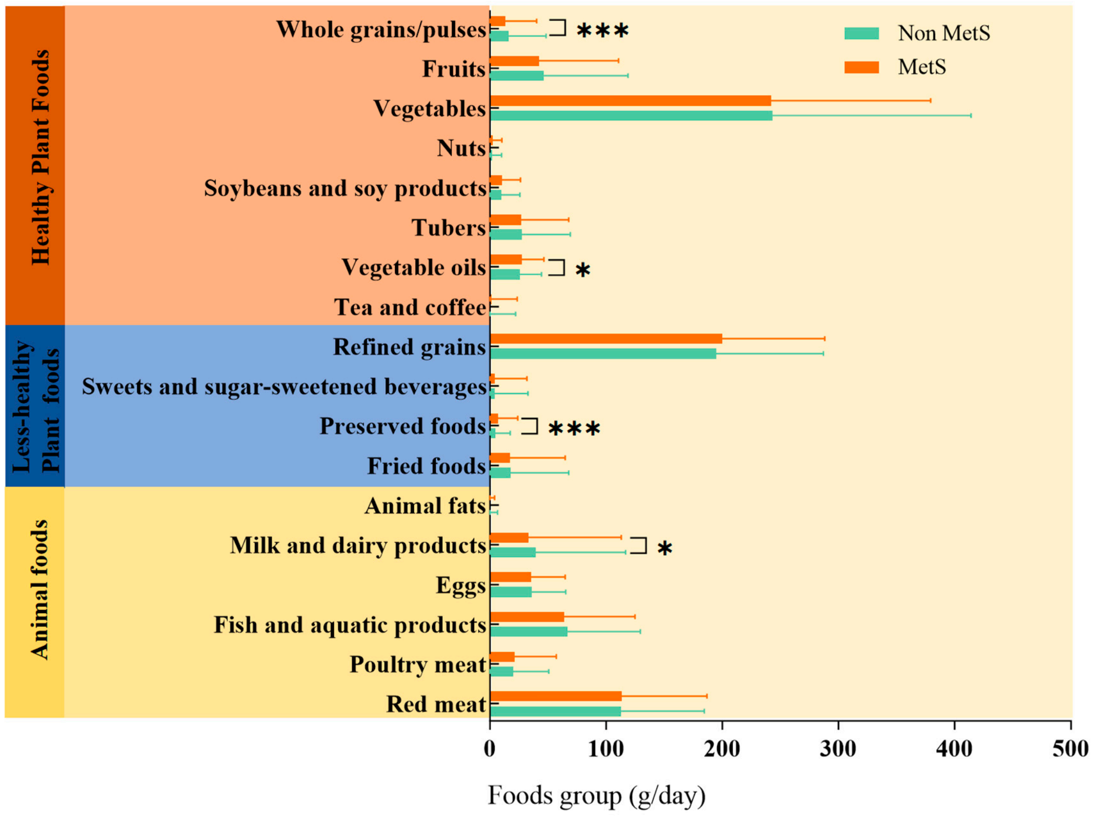

Statistical significance markers (*P < 0.05; ***P < 0.001) denote between-group differences when comparing the dietary intake levels of 18 distinct food categories between Non-Metabolic Syndrome (Non-Mets) and Metabolic Syndrome (Mets) groups.

Table S1. Plant-based Diet Indices Construction, Scoring, and Food Items

Food Category	Food Groups	Food Items	PDI	hPDI	uPDI
Healthy plant Foods	Whole grains/pulses*	Wheat, Oatmeal/Oats, Brown Rice, Black Rice, Corn, Barley, Millet, Quinoa, Rye, Buckwheat, Red Beans, Mung Beans, Chickpeas, Kidney Beans, Lentils, Dried Peas, Black-eyed Peas	Positive	Positive	Reverse
	Fruits	Bayberry, Loquat, Peach, Satsuma Mandarin, Kyoho Grapes, Strawberry, Persimmon, Green Plum, Apple, Banana, Watermelon, Orange, Pear, Kiwi, Plum	Positive	Positive	Reverse
	Vegetables	Dark-colored Fresh Vegetables (e.g. Spinach, Water Spinach, Amaranth) Light-colored Vegetables (e.g. Chinese Cabbage, Lettuce, Indian Lettuce)	Positive	Positive	Reverse
	Nuts	Walnut, Almond, Cashew, Peanut, Pumpkin Seed	Positive	Positive	Reverse
	Soybeans and soy products	Yellow Soybean ,Green Soybean, Edamame (Fresh Soybean), Soymilk, Tofu, Tofu Pudding, Soybean Meal	Positive	Positive	Reverse
	Tubers	Potato, Sweet potato, Purple sweet potato, Yam, Cassava, Taro,			
	Vegetable oils	Soybean oil, Rapeseed oil, Peanut oil, Corn oil, Sunflower seed oil, Blended oil, Olive oil			
	Tea and coffee	Coffee, tea	Positive	Positive	Reverse
Unhealthy plant foods	Refined grains	White rice, White flour, Refined noodles, Refined rice flour, Rice cake, White rice steamed bun, bread	Positive	Reverse	Positive
	Sweets and sugar-sweetened beverages	Carbonated beverages (Cola, Sprite, Jianlibao), Solid beverages (milk tea powder), Cakes and desserts (Chinese mooncakes, mung bean cakes, tangyuan; Western cakes,	Positive	Reverse	Positive

		pudding, macarons), Sugars (white sugar, brown sugar, rock sugar)			
	Preserved foods	Pickled vegetables, Pickled vegetables in bean sauce (cucumber in bean sauce, radish in bean sauce, eight-treasure pickled vegetables),Fermented bean curd	Positive	Reverse	Positive
	Fried foods	Youtiao (deep-fried dough sticks), Mahua (twisted dough twists), Fried chicken drumsticks, French fries, Youbing (deep-fried dough cake), fried instant noodles	Positive	Reverse	Positive
Animal foods	Animal fat	Lard (rendered from pig fatback/pork belly), Beef tallow, Mutton fat, butter/margarine	Reverse	Reverse	Reverse
	Milk and dairy products	Milk, Yogurt, Cheese,	Reverse	Reverse	Reverse
	Egg	Chicken egg, Duck egg, Goose egg, Quail egg, Preserved egg (Songhua egg)	Reverse	Reverse	Reverse
	Fish and aquatic products	Fish (freshwater & saltwater), Shrimp, Crab, Shellfish, Cephalopods (squid/octopus), Processed products (dried fish/fish balls), Mollusks (abalone/oyster), Coelenterates (jellyfish)	Reverse	Reverse	Reverse
	Poultry meat	Chicken meat, Duck meat, Goose meat	Reverse	Reverse	Reverse
	Red meat	Beef, Pork, Mutton	Reverse	Reverse	Reverse

*Whole Grains/Pulses: "Pulses" refers to legumes like lentils, chickpeas, etc., excluding soybeans. The PDI, hPDI, and uPDI categorized food groups into “healthy plant foods,” “less-healthy plant foods,” and “animal foods.” PDI, overall plant-based diet index; hPDI, healthful plant-based diet index; uPDI, unhealthful plant-based diet index.

Table S2 Characteristics of 18 Plant-Based Dietary Components in Metabolic Syndrome

Variables	Total (n = 4695)	Non MetS (n = 3573)	MetS (n = 1122)	Statistic	P
Healthy Plant Foods group(g/day)					
Whole grains/pulses*	15.54 ± 30.91	16.28 ± 31.97	13.17 ± 27.13	t=3.20	0.001
Fruits	45.53 ± 71.39	46.52 ± 72.32	42.37 ± 68.30	t=1.70	0.089
Vegetables	243.16 ± 163.18	243.42 ± 170.62	242.34 ± 136.91	t=0.19	0.847
Nuts	2.30 ± 7.95	2.26 ± 7.94	2.44 ± 7.98	t=-0.65	0.518
Soybeans and soy products	10.23 ± 15.68	10.20 ± 15.55	10.30 ± 16.11	t=-0.18	0.857
Tubers	27.29 ± 41.54	27.49 ± 41.65	26.67 ± 41.19	t=0.58	0.563
Vegetable oils	26.39 ± 18.51	26.04 ± 18.35	27.51 ± 18.97	t=-2.32	0.020
Tea and coffee	1.16 ± 21.35	1.10 ± 21.05	1.35 ± 22.29	t=-0.35	0.726
Less-healthy Plant Foods group(g/day)					
Refined grains	196.10 ± 91.15	194.93 ± 92.00	199.80 ± 88.32	t=-1.56	0.119
Sweets and sugar-sweetened beverages	4.58 ± 27.96	4.63 ± 28.10	4.42 ± 27.52	t=0.22	0.826
Preserved foods	5.48 ± 13.79	5.02 ± 12.52	6.94 ± 17.13	t=-3.47	<0.001
Fried foods	17.95 ± 49.19	18.07 ± 49.81	17.56 ± 47.18	t=0.30	0.761
Animal foods group(g/day)					
Animal fats	0.71 ± 5.39	0.76 ± 5.85	0.55 ± 3.56	t=1.11	0.267
Milk and dairy products	37.83 ± 78.12	39.24 ± 77.59	33.34 ± 79.67	t=2.18	0.030
Eggs	36.21 ± 28.89	36.30 ± 28.87	35.92 ± 28.95	t=0.39	0.696
Fish and aquatic products	65.98 ± 62.51	66.62 ± 63.00	63.94 ± 60.91	t=1.25	0.211
Poultry meat	20.58 ± 31.69	20.28 ± 30.36	21.51 ± 35.59	t=-1.13	0.260
Red meat	112.95 ± 72.09	112.84 ± 71.62	113.30 ± 73.60	t=-0.19	0.850

*Whole Grains/Pulses: "Pulses" refers to legumes like lentils, chickpeas, etc., excluding soybeans.

Table S3: Associations between PDI, hPDI, and MetS (N=4,695)

	Q1	Q2	Q3	Q4	Q5	*P*-trend
				PDI		
Model1	Ref	0.97 (0.78 ~ 1.20)	0.98 (0.78 ~ 1.22)	1.01 (0.81 ~ 1.24)	1.09 (0.88 ~ 1.34)	0.355
Model2	Ref	0.95 (0.77 ~ 1.18)	0.96 (0.76 ~ 1.20)	0.98 (0.79 ~ 1.22)	1.02 (0.82 ~ 1.26)	0.756
Model3	Ref	0.91 (0.72 ~ 1.14)	0.89 (0.70 ~ 1.12)	0.91 (0.73 ~ 1.14)	0.98 (0.78 ~ 1.22)	0.939
				hPDI		
Model1	Ref	0.96 (0.77 ~ 1.21)	1.00 (0.81 ~ 1.24)	0.94 (0.77 ~ 1.16)	0.94 (0.76 ~ 1.16)	0.510
Model2	Ref	0.97 (0.77 ~ 1.21)	1.01 (0.81 ~ 1.25)	0.93 (0.75 ~ 1.14)	0.92 (0.74 ~ 1.14)	0.932
Model3	Ref	0.92 (0.72 ~ 1.16)	0.99 (0.79 ~ 1.24)	0.90 (0.73 ~ 1.12)	0.93 (0.74 ~ 1.16)	0.479

Model 1 was unadjusted; Model 2 adjusted for age and sex; and Model 3 further adjusted for ethnicity, region, family history of diabetes and hypertension, smoking status, alcohol status, takeaway food consumption, physical activity level, education level, total energy intake, and BMI residuals.
